# Hypertension and heart failure: Navigating blood pressure targets and medication titration and tolerance

**DOI:** 10.1016/j.clinme.2026.100584

**Published:** 2026-04-17

**Authors:** Irfan Helmy, Mohamad Karnib, Varun Sundaram

**Affiliations:** a Case Western Reserve University School of Medicine, Cleveland, OH, USA; b Department of Cardiology, Louis Stokes Cleveland Veterans Affairs Medical Center, Cleveland, OH, USA; c University Hospitals Cleveland Medical Center, Cleveland, OH, USA

**Keywords:** Hypertension, Heart failure, Blood pressure targets, Guideline-directed medical therapy, Heart failure with reduced ejection fraction, Heart failure with preserved ejection fraction

## Abstract

This article briefly reviews the latest hypertension guidelines and how they should be individualised in patients with heart failure (HF), including the two specific phenotypes: HF with reduced ejection fraction (HFrEF) and HF with preserved ejection fraction (HFpEF). Key recent trials and guidelines show that HF medications remain beneficial and safe to use even in patients without overt hypertension, provided that they are tolerated. We also outline practical strategies for titration and selection of HF medications, most of which affect BP, in an outpatient setting. These strategies are applicable to both normotensive patients and those with pre-existing hypertension.

## Graphical Abstract

**Figure:**
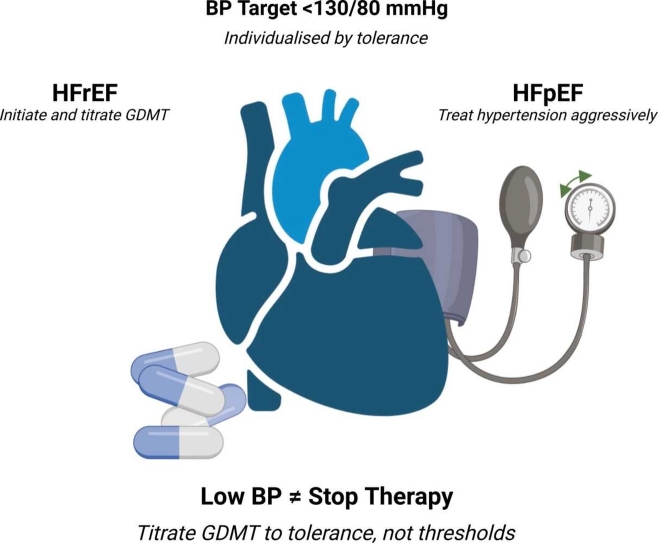
Created in BioRender. Helmy, I. (2026) https://BioRender.com/3orgxo4

## Introduction

Hypertension and heart failure (HF) are closely intertwined. Long-standing hypertension is a major independent risk factor for developing HF, and uncontrolled high blood pressure (BP) accelerates progression of HF.^1^ Conversely, many first-line guideline-directed medical therapies (GDMT) for heart failure are antihypertensive drugs, meaning that clinicians often manage both conditions simultaneously.

This article reviews the latest hypertension guidelines and their individualisation in HF, including HF with reduced ejection fraction (HFrEF) and HF with preserved ejection fraction (HFpEF). Key recent trials and guidelines show that HF medications remain beneficial and safe to use even in patients without overt hypertension, provided that they are tolerated. We also outline practical outpatient strategies for titration and selection of HF medications, including in normotensive patients and those with pre-existing hypertension.

## Updated blood pressure guidelines

American and European guidelines have strengthened recommendations for lower BP targets to reduce cardiovascular morbidity and mortality. The 2025 American Heart Association/American College of Cardiology (AHA/ACC) Hypertension Guidelines continue to recommend a treatment target of <130/80 mmHg for adults, regardless of their baseline cardiovascular risk.^2^ A large meta-analysis showed that every 10 mmHg reduction in systolic BP was associated with a 28% lower risk of heart failure (in addition to a 27% lower stroke risk, 17% lower risk of coronary heart disease and 13% reduced all-cause mortality).^3^ These findings were seen independent of baseline systolic BP, including in those with systolic BP <130 mmHg. The SPRINT trial recruited participants without diabetes but at high atherosclerotic cardiovascular risk, and treating to a systolic BP of ∼120 mmHg compared to ∼135 mmHg resulted in a 38% reduction in incident heart failure over a median follow-up of 3.3 years.^4^

The 2024 European Society of Cardiology/European Society of Hypertension (ESC/ESH) guidelines similarly recommend a target of <130/80 mmHg as well, but allow for more liberalised targets (<140/90 mmHg) in specific clinical scenarios, such as those with pre-existing orthostatic hypotension, advanced age (>85 years), or an anticipated life expectancy of less than 3 years.^5^ The 2019 NICE Hypertension guidelines adopt a more individualised approach, tailoring BP targets on patient age and underlying cardiovascular disease risk, with targets ranging from 130/80 to 150/90 mmHg (Table 1).^6^

**Table 1:** Recommended blood pressure targets by patient population across major international guidelines.

Guideline	Patient population	BP target
2025 ACC/AHA^2^	Adults	<130/80 mmHg
2024 ESC/ESH^5^	Age >85 years, pre-existing orthostatic hypotension, life expectancy <3 years	<140/90 mmHg
	All other adults	<130/80 mmHg
2019 NICE^6^	Age over 80 with hypertension or type 1 diabetes	<150/90 mmHg
	1. Age >80 with CKD and urine ACR <70 mg/mmol 2. Adults aged under 80 with hypertension	<140/90 mmHg
	1. Age >80 with CKD plus urine ACR >70 mg/mmol 2. Age <80 with type 1 diabetes and urine ACR >70 mg/mmol 3. Age <80 with CKD and urine ACR >70 mg/mmol	<130/90 mmHg

## Initiating guideline-directed medical therapies in HFrEF: ‘low’ BP is not a contraindication

Pharmacologic therapy is the cornerstone of HFrEF management. Both the 2021 ESC and the 2022 AHA/ACC/HFSA guidelines strongly endorse the four ‘pillars’ of GDMT: beta-blockers, renin–angiotensin system inhibitors (angiotensin-converting enzyme inhibitors [ACEi], angiotensin II receptor blockers [ARB], and angiotensin receptor-neprilysin inhibitor [ARNI]), mineralocorticoid receptor antagonists (MRA), and sodium-glucose cotransporter-2 inhibitors (SGLT2i), each with a Class I recommendation.^7^, ^8^ Individually, but especially collectively, they reduce mortality, HF hospitalisations, and improve long-term symptom burden. In fact, when considered collectively, contemporary HF drugs yield a remarkably low number needed to treat, approximately four to five patients, to prevent one death, underscoring the profound survival benefit that these agents confer at the population level.^9^

A common clinical concern is whether it is safe to start or uptitrate these medications in those who have ‘low’ to normal blood pressures (eg systolic BP 95–120 mmHg). Landmark HFrEF trials consistently demonstrate that it is both safe and beneficial to initiate these drugs in patients with low to normal blood pressures, provided that individuals are asymptomatic and closely monitored for symptoms post-initiation. In fact, most HFrEF trials randomised participants with systolic BPs >95–100 mmHg, and showed strong clinical benefits without excessive hypotension-related adverse events or major increases in discontinuation. Among these therapies, SGLT2 inhibitors have only a minimal BP-lowering effect, and therefore should not usually be withheld solely because baseline BP is low to normal.^10^, ^11^ Key HFrEF trials, BP parameters and discontinuation rates are summarised in Table 2: Panel A.

**Table 2:** Blood pressure criteria and tolerability in landmark heart failure trials.

Panel A: heart failure with reduced ejection fraction							
Drug class	Trial	Intervention (vs. control)	Exclusion SBP	Mean SBP at time of randomisation (mmHg)	Mean SBP change (mmHg)	Hypotension	Drug Discontinuation
**SGLT2-inhibitor**	**DAPA-HF**^10^	Dapagliflozin 10 mg daily vs. placebo	<95 mmHg, or **symptomatic** hypotension	122.0±16.3	−1.92± 14.92	0.3% vs. 0.5%	4.7% vs. 4.9%
**SGLT2-inhibitor**	**EMPEROR-Reduced**^11^	Empagliflozin 10 mg daily vs. placebo	<100 mmHg, or **symptomatic** hypotension	122.6±15.9	−2.4±0.4	Any: 9.4% vs. 8.7% Symptomatic: 5.7% vs. 5.5%	16.3% vs. 18.0%
**Angiotensin-Receptor Neprilysin Inhibitor**	**PARADIGM-HF**^12^	Sacubitril-valsartan (titrated) vs. enalapril 10 mg BID	<95 mmHg or **symptomatic** hypotension	122±15	Modest reduction^a^	Symptomatic: 14.0% vs. 9.2% Symptomatic with SBP <90: 2.7% vs. 1.4%	17.8% vs. 19.8%
**Angiotensin Converting Enzyme Inhibitor**	**SOLVD**^13^	Enalapril (titrated) vs. placebo	No predefined SBP exclusion	125.3/77.3	−4.7 vs. 4.0	Dizziness: 57% vs. 50%	32.5% vs. 41.4%
**Mineralocorticoid Receptor Antagonist**	**RALES**^14^	Spironolactone 25–50 mg vs. placebo	No predefined SBP exclusion	123±21/75±12	Not systematically reported	Not systematically reported	8% vs. 5%
**Beta Blocker**	**COPERNICUS**^15^	Carvedilol (titrated) vs. placebo	<85 mmHg	123±19/76±11	Not systematically reported	Not systematically reported	14.8% vs. 18.5%

Panel B: heart failure with preserved ejection fraction						
Trial	Intervention (vs. control)	Exclusion SBP	Mean SBP at time of randomisation (mmHg)	Mean SBP change (mmHg)	Hypotension	Drug discontinuation
**EMPEROR-Preserved**^16^	Empagliflozin 10 mg daily vs. placebo	<100 mmHg, or **symptomatic** hypotension	131.8±15.6	−1.8±0.3	Symptomatic: 6.6% vs. 5.2%	19.1% vs. 18.4%
**PARAGON**^17^	Sacubitril-valsartan (titrated) vs. valsartan	<100 mmHg, or **symptomatic** hypotension	130.5±15.6	Modest reduction^a^	SBP <100 mmHg: 15.8% vs. 10.8%	25.3% vs. 26.7%
**TOPCAT**^18^	Spironolactone (titrated) vs. placebo	Orthostatic hypotension. No predefined SBP exclusion	130/80	−2.7 vs. −0.2	Not systematically reported	34.3% vs. 31.4%

All definitions were per trials’ protocol. Comparisons are reported as study drug vs. comparator. Most trials randomised patients with baseline SBP ∼120 mmHg and demonstrated low rates of treatment discontinuation due to hypotension. Abbreviations: AE, adverse event; BID, twice daily; BP, blood pressure; SBP, systolic blood pressure; DBP, diastolic blood pressure; HFrEF, heart failure with reduced ejection fraction.

a Sacubitril-valsartan was associated with greater early SBP reduction than enalapril, with attenuation over time.

In these trials and in clinical practice, GDMT dosing is guided by patient tolerability, with symptomatic hypotension rather than an isolated BP threshold being the key reason to consider down-titration or withdrawal. In practice, initiation and uptitration of GDMT should also be informed by HF patient profiling, incorporating heart rate, congestion status, renal function, and other markers of tolerability to better tailor therapy to the individual patient.^19^ Some trials used an aggressive inclusion criterion of systolic BP as low as 90 mmHg, provided the patient was asymptomatic at baseline.^20^ Titration protocols in these trials, as well as in several experts’ opinions, called for gradually increasing the medication dose as long as the patient remained stable: no symptomatic hypotension (ie dizziness or syncope), bradycardia or other limiting side effects (eg hyperkalaemia or renal dysfunction for renin–angiotensin–aldosterone system inhibitors).^20^ Although clinical hesitation in starting and uptitrating these heart failure drugs in the context of low or low to normal BP is understandable, an asymptomatic BP reduction alone should not be a barrier to initiation or continuation of these lifesaving HF drugs.

## HFpEF management

Hypertension is present in the majority of patients with HFpEF and represents one of the most important identified causes of this syndrome. Longstanding hypertension progresses from early diastolic dysfunction to concentric LV hypertrophy, arterial stiffness, microvascular dysfunction and fibrosis, a pattern most often associated with HFpEF and hypertensive acute HF, whereas later chamber dilation and systolic dysfunction may lead to HFrEF and support phenotype-specific treatment.^21^

Current guidelines recommend treating hypertension in HFpEF, with target blood pressures generally <130/80 mmHg. This recommendation is largely extrapolated from the broader hypertension literature, as trials specifically testing BP-lowering strategies in established hypertensive HFpEF patients are lacking.^2^, ^5^, ^22^ However, the SPRINT trial showed reductions in cardiovascular events, even in the subgroup with clinical cardiovascular disease, by targeting a systolic BP of ∼120 mmHg.^4^

Nevertheless, the optimal lower BP target in HFpEF remains uncertain. Observational data suggest a possible J-shaped relationship between BP and outcomes, with increased risk at systolic pressures <120 mmHg, and intensive lowering may increase the risk of renal dysfunction. Notably, current guidelines do not specify a lower BP threshold at which therapy should be reduced or discontinued.^2^, ^5^

Most patients with HFpEF and hypertension require combination therapy to achieve BP control. Therapies with proven benefit in HFpEF, SGLT2i and MRAs (Table 2: Panel B) should be incorporated, although SGLT2 inhibitors have only a modest effect on BP.^16^ Other agents should be selected by comorbidities and tolerability, with beta-blockers and nitrates generally avoided unless specifically indicated.^22^

## Persistent hypertension in heart failure

While many patients with HF have normal or low to normal BP once on therapy, some individuals (particularly those with earlier-stage disease or HFpEF) will remain hypertensive despite guideline-recommended doses of HF medications. The 2021 ESC guidelines recommend uptitrating HFrEF GDMT to maximally tolerated doses (Table 3).^7^ Higher doses of GDMT have been associated with improved outcomes. For example, higher carvedilol doses reduce mortality and HF hospitalisations, and higher-dose lisinopril is associated with fewer HF hospitalisations in HFrEF.^7^ The guideline-recommended target doses and common reasons for intolerance are summarised in Table 3.^8^

**Table 3:** Guideline-recommended doses for guideline-directed medical therapy for heart failure with reduced ejection fraction and common reasons for intolerance.^8^

Drug	Guideline-recommended dose	Reasons for intolerance
Metoprolol succinate	200 mg once daily	Symptomatic hypotension, bradycardia or heart block, functionally-limiting fatigue
Carvedilol	25–50 mg twice daily	
Bisoprolol	10 mg once daily	
Lisinopril	20–40 mg once daily	Symptomatic hypotension, hyperkalaemia, renal dysfunction, angioedema
Losartan	50–150 mg once daily	
Sacubitril-Valsartan	97–103 mg twice daily	
Spironolactone	50 mg once daily	Hyperkalaemia
Empagliflozin	10 mg once daily	Orthostasis, mycotic genital infection, prior Fournier’s gangrene
Isosorbide dinitrate and hydralazine	120 mg isosorbide and 300 mg hydralazine total daily in divided doses	Orthostasis, headache, poor adherence due to frequent dosing

**Fig. 1:**
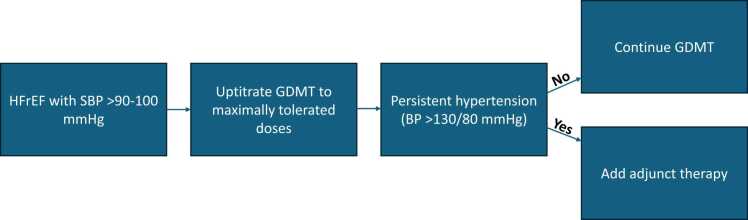
Suggested approach to blood pressure management in patients with heart failure with reduced ejection fraction (HFrEF). Guideline-directed medical therapy (GDMT) should be optimised to maximally tolerated doses prior to adding adjunctive antihypertensive agents. Abbreviations: SBP, systolic blood pressure; GDMT, guideline-directed medical therapy; BP, blood pressure.

Acute BP goals in decompensated HF differ from chronic outpatient targets, with the former focused on stabilisation and decongestion, and intravenous vasodilators may be useful in selected patients with hypertensive acute pulmonary oedema. Once clinically stabilised, patients should be transitioned to chronic oral therapy.^23^ If BP remains above target despite maximally tolerated GDMT, additional antihypertensive therapy should be selected, based on the HF phenotype and safety profile. In HFrEF, one option is the combination of hydralazine and nitrates, which has shown benefit in some HFrEF populations, through limited by thrice-daily dosing.^8^ The 2021 ESC guidelines suggest amlodipine or felodipine as they have shown to be safe in HFrEF, but medications such as non-dihydropyridine calcium channel blockers (eg diltiazem and verapamil), alpha-blockers and centrally acting agents such as moxonidine should be avoided given their association with worse outcomes in HFrEF.^7^ Ultimately, the goal is to achieve the BP targets set by the guidelines (generally <130/80 mmHg) even in patients with HF, to reduce long-term cardiovascular risk (Fig. 1).

## Conclusions and take-home messages

Hypertension and HF management remain inseparable in current clinical practice. Recent evidence and guidelines have refined our approach to both. Recent guidelines emphasise stricter BP targets (generally <130/80 mmHg) to prevent adverse cardiovascular outcomes such as HF. For clinicians caring for patients with HF, a few key points should be kept in mind:

1. *First, GDMT for HFrEF should not be withheld solely because BP is ‘low to normal’*. Most landmark HFrEF trials enrolled patients with systolic BP of 95–130 mmHg. Initiation of these agents demonstrated substantial morbidity and mortality benefits, with only modest additional reductions in BP and low rates of symptomatic hypotension.
2. *Second, uptitration of medications should be guided by clinical tolerability rather than arbitrary BP cut-offs*. In HFrEF, the goal is to achieve target or maximally tolerated doses (Table 3), provided that patients remain asymptomatic and adverse reactions are manageable.
3. *Third, persistent hypertension should be viewed as an opportunity to optimise GDMT, rather than as an indication to add non-HF-specific antihypertensive agents*. Maximising tolerated doses of evidence-based HF therapies should remain the top priority.
4. *Finally, adjunct antihypertensives should be selected thoughtfully.* In patients with HFrEF and persistent hypertension despite maximal doses of GDMT, choose agents that are proven to be safe (eg hydralazine, nitrates), and avoid drugs associated with harm (eg diltiazem, verapamil). In HFpEF, standard antihypertensive regimens can be used more liberally, alongside therapies with HFpEF-specific benefit (eg MRA).

## CRediT authorship contribution statement

**Mohamad Karnib:** Writing – review & editing. **Varun Sundaram:** Writing – review & editing, Supervision, Methodology, Conceptualization. **Irfan Helmy:** Writing – review & editing, Writing – original draft, Visualization, Investigation, Conceptualization.

## Funding

This research did not receive any specific grant from funding agencies in the public, commercial or not-for-profit sectors.

## Declaration of competing interest

The authors declare that they have no known competing financial interests or personal relationships that could have appeared to influence the work reported in this paper.

## References

[1] Kannan A., Janardhanan R. (2014). Hypertension as a risk factor for heart failure. Curr Hypertens Rep.

[2] Writing Committee Members*, Jones D.W., Ferdinand K.C. (2025). 2025 AHA/ACC/AANP/AAPA/ABC/ACCP/ACPM/AGS/AMA/ASPC/NMA/PCNA/SGIM guideline for the prevention, detection, evaluation and management of high blood pressure in adults: a report of the American College of Cardiology/American Heart Association Joint Committee on clinical practice guidelines. Hypertension.

[3] Ettehad D., Emdin C.A., Kiran A. (2016). Blood pressure lowering for prevention of cardiovascular disease and death: a systematic review and meta-analysis. Lancet.

[4] SPRINT Research Group, Wright J.T., Williamson J.D. (2015). A randomized trial of intensive versus standard blood-pressure control. N Engl J Med.

[5] 2024 ESC guidelines for the management of elevated blood pressure and hypertension. Eur Heart J. Oxford Academic. 〈https://academic.oup.com/eurheartj/article/45/38/3912/7741010?login=true〉 Accessed December 7, 2025.

[6] Recommendations. Hypertension in adults: diagnosis and management. Guidance. NICE; 2019. 〈https://www.nice.org.uk/guidance/ng136/chapter/Recommendations〉 Accessed December 7, 2025.

[7] McDonagh T.A., Metra M., Adamo M. (2021). 2021 ESC guidelines for the diagnosis and treatment of acute and chronic heart failure: developed by the Task Force for the diagnosis and treatment of acute and chronic heart failure of the European Society of Cardiology (ESC) with the special contribution of the Heart Failure Association (HFA) of the ESC. Eur Heart J.

[8] Heidenreich P.A., Bozkurt B., Aguilar D. (2022). 2022 AHA/ACC/HFSA guideline for the management of heart failure: a report of the American College of Cardiology/American Heart Association Joint Committee on clinical practice guidelines. Circulation.

[9] Greene S.J., Ayodele I., Pierce J.B. (2024). Eligibility and projected benefits of rapid initiation of quadruple therapy for newly diagnosed heart failure. JACC Heart Fail.

[10] McMurray J.J.V., Solomon S.D., Inzucchi S.E. (2019). Dapagliflozin in patients with heart failure and reduced ejection fraction. N Engl J Med.

[11] Packer M., Anker S.D., Butler J. (2020). Cardiovascular and renal outcomes with empagliflozin in heart failure. N Engl J Med.

[12] Angiotensin–neprilysin inhibition versus enalapril in heart failure. N Engl J Med. 〈https://www.nejm.org/doi/full/10.1056/NEJMoa1409077〉 Accessed December 11, 2025. 10.1056/NEJMoa1409077 25176015

[13] SOLVD Investigators, Yusuf S., Pitt B., Davis C.E., Hood W.B., Cohn J.N. (1991). Effect of enalapril on survival in patients with reduced left ventricular ejection fractions and congestive heart failure. N Engl J Med.

[14] Pitt B., Zannad F., Remme W.J. (1999). The effect of spironolactone on morbidity and mortality in patients with severe heart failure. Randomized Aldactone Evaluation Study Investigators. N Engl J Med.

[15] Packer M., Coats A.J.S., Fowler M.B. (2001). Effect of carvedilol on survival in severe chronic heart failure. N Engl J Med.

[16] Anker S.D., Butler J., Filippatos G. (2021). Empagliflozin in heart failure with a preserved ejection fraction. N Engl J Med.

[17] Solomon S.D., McMurray J.J.V., Anand I.S. (2019). Angiotensin–neprilysin inhibition in heart failure with preserved ejection fraction. N Engl J Med.

[18] Pitt B., Pfeffer M.A., Assmann S.F. (2014). Spironolactone for heart failure with preserved ejection fraction. N Engl J Med.

[19] Rosano G.M.C., Moura B., Metra M. (2021). Patient profiling in heart failure for tailoring medical therapy. A consensus document of the Heart Failure Association of the European Society of Cardiology. Eur J Heart Fail.

[20] Clinical management and therapeutic optimization of patients with heart failure with reduced ejection fraction and low blood pressure. A clinical consensus statement of the Heart Failure Association (HFA) of the ESC - Skouri - 2025. Eur J Heart Fail. Wiley Online Library. 〈https://onlinelibrary.wiley.com/doi/10.1002/ejhf.3618〉 Accessed December 10, 2025. 10.1002/ejhf.3618 40012353

[21] Geavlete O., Collins S.P., Mebazaa A. (2025). Hypertensive acute heart failure: a critical perspective on definition, epidemiology, pathophysiology, and prognosis—a narrative review: a joint session with the Romanian Society of Cardiology (part II). Heart Fail Rev.

[22] Kittleson M.M., Panjrath G.S., Amancherla K. (2023). 2023 ACC expert consensus decision pathway on management of heart failure with preserved ejection fraction: a report of the American College of Cardiology Solution Set Oversight Committee. J Am Coll Cardiol.

[23] Chioncel O., Mebazaa A., Farmakis D. (2025). Pathophysiology and clinical use of agents with vasodilator properties in acute heart failure. A scientific statement of the Heart Failure Association (HFA) of the European Society of Cardiology (ESC). Eur J Heart Fail.

